# Supplementary material for: Estimating total human deaths from rabies: A statistical framework leveraging reports of rabies transmission from deceased organ and tissue donors
Source: PLoS Negl Trop Dis. 2026 Sep 21;20(9):e0014731. doi: 10.1371/journal.pntd.0014731 (PMC13592719; doi:10.1371/journal.pntd.0014731)
Supplement: Supplementary file 6 [file pntd.0014731.s005.zip › Result_B/China/Figure_S3_China.pdf]

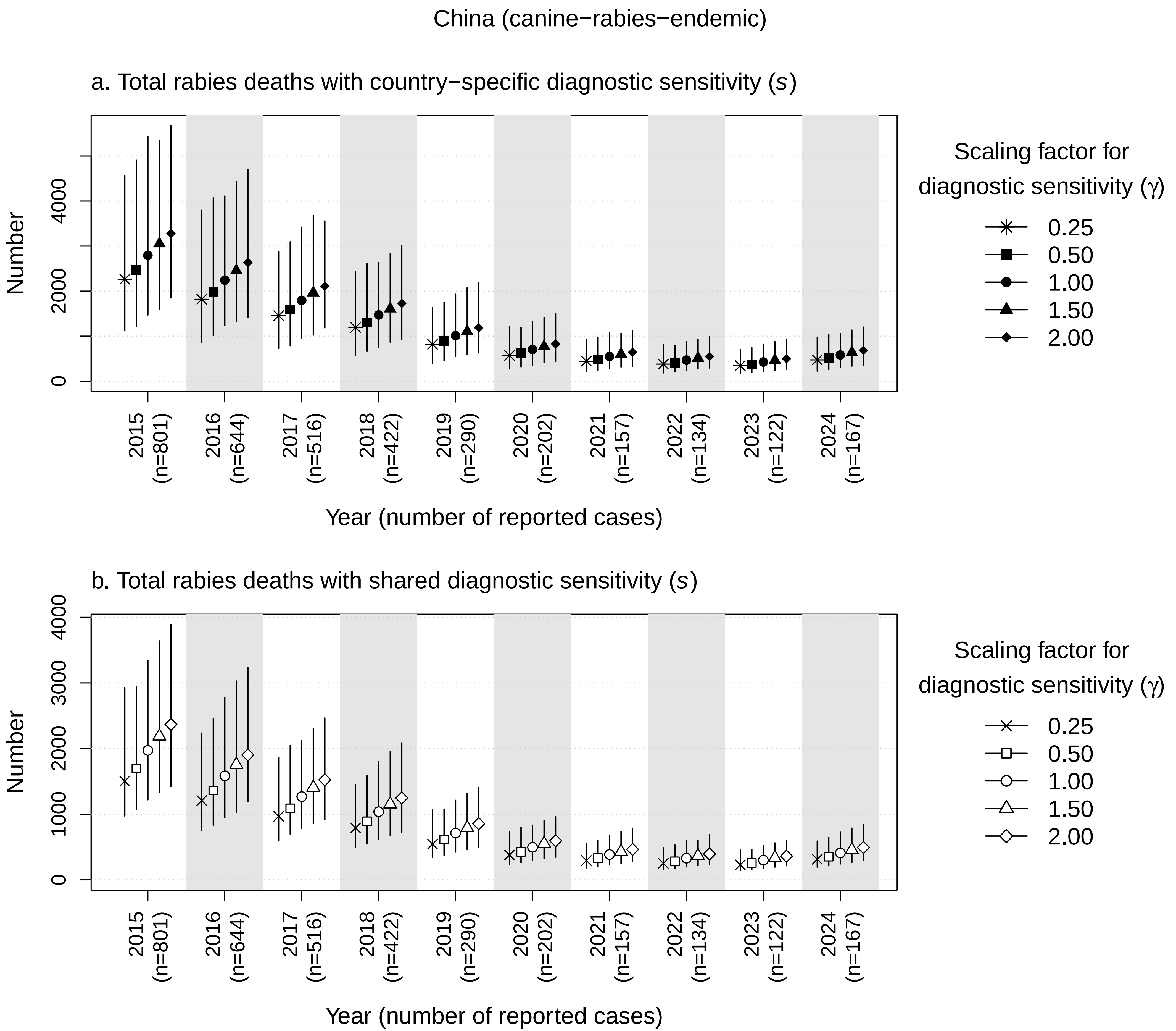

China (canine−rabies−endemic)
a. Total rabies deaths with country−specific diagnostic sensitivity (s)
Number
0
2000
4000
2015 (n=801)
2016 (n=644)
2017 (n=516)
2018 (n=422)
2019 (n=290)
2020 (n=202)
2021 (n=157)
2022 (n=134)
2023 (n=122)
2024 (n=167)
Year (number of reported cases)
Scaling factor for diagnostic sensitivity (γ)
0.25
0.50
1.00
1.50
2.00
b. Total rabies deaths with shared diagnostic sensitivity (s)
Number
0
1000
2000
3000
4000
2015 (n=801)
2016 (n=644)
2017 (n=516)
2018 (n=422)
2019 (n=290)
2020 (n=202)
2021 (n=157)
2022 (n=134)
2023 (n=122)
2024 (n=167)
Year (number of reported cases)
Scaling factor for diagnostic sensitivity (γ)
0.25
0.50
1.00
1.50
2.00
